# Implications the Role of miR-155 in the Pathogenesis of Autoimmune Diseases

**DOI:** 10.3389/fimmu.2021.669382

**Published:** 2021-05-07

**Authors:** Salar Pashangzadeh, Morteza Motallebnezhad, Fatemeh Vafashoar, Azadeh Khalvandi, Nazanin Mojtabavi

**Affiliations:** Department of Immunology, School of Medicine, Iran University of Medical Sciences, Tehran, Iran

**Keywords:** miRNA, miR-155, autoimmune disease, immune system, tolerance

## Abstract

MicroRNAs (miRNAs) are small noncoding conserved RNAs containing 19 to 24 nucleotides that are regulators of post-translational modifications and are involved in the majority of biological processes such as immune homeostasis, T helper cell differentiation, central and peripheral tolerance, and immune cell development. Autoimmune diseases are characterized by immune system dysregulation, which ultimately leads to destructive responses to self-antigens. A large body of literature suggests that autoimmune diseases and immune dysregulation are associated with different miRNA expression changes in the target cells and tissues of adaptive or innate immunity. miR-155 is identified as a critical modulator of immune responses. Recently conducted studies on the expression profile of miR-155 suggest that the altered expression and function of miR-155 can mediate vulnerability to autoimmune diseases and cause significant dysfunction of the immune system.

## Introduction

**Figure 1:**
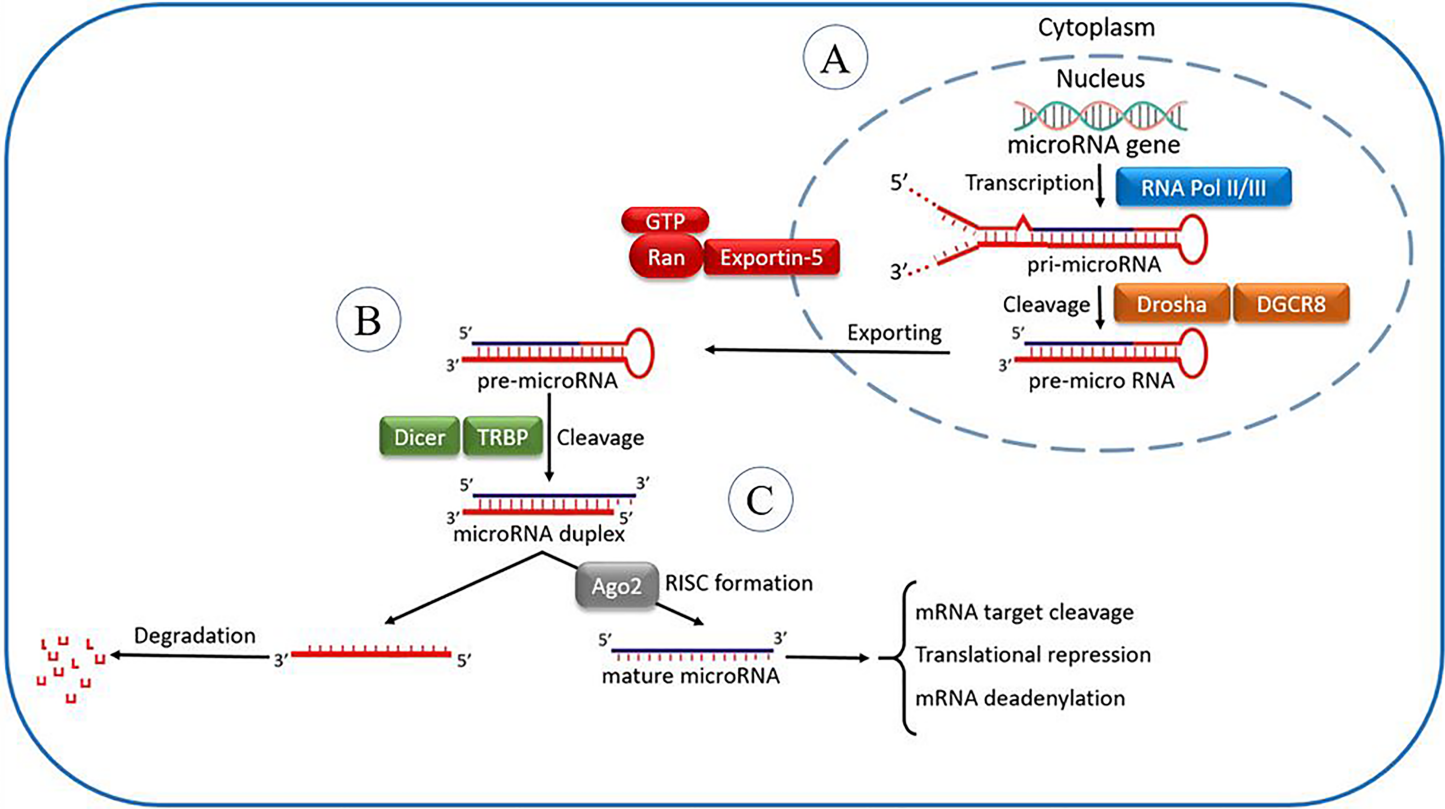
The microRNA processing pathway. It is postulated that the miRNA processing pathway is linear and universal to all mammalian miRNAs. This process includes the production of the primary miRNA transcript (pri-miRNA) by RNA polymerase II or III and cleavage of the pri-miRNA by the microprocessor complex Drosha–DGCR8 (Pasha) in the nucleus **(A)**. The resulting precursor hairpin, the pre-miRNA, is exported from the nucleus by Exportin-5–Ran-GTP. In the cytoplasm, the RNase Dicer in complex along with RNA-binding cofactor of Dicer complexes known as transactivation response element RNA-binding protein (TRBP) cleaves the pre-miRNA hairpin to its mature length **(B)**. The mature miRNA’s functional strand is loaded together with Argonaute (Ago2) proteins into the RNA-induced silencing complex (RISC), where it guides RISC to silence target mRNAs through mRNA cleavage, translational repression, or deadenylation, whereas the passenger strand is degraded **(C)**.

Understanding miRNAs’ role opened a new aspect of discovering disease pathogenesis and conferred a targeted therapy for a diverse spectrum of diseases. miRNAs are small noncoding conserved RNAs, with a length of 19 to 24 nucleotides and regulators of post-translational modifications (1, 2). Specific animal and human studies discovered various roles of miRNAs and their mechanism of action. It is known that mature miRNAs interact with definite messenger RNAs (mRNAs) to repress gene expression. Usually, the target mRNA is identified by the ‘seed’ region of miRNAs, which consist of 2–7 nucleotides (3). In the case of complementary base pairing matching or semi matching, the induction of endonuclease cleavage occurs, which causes the degradation of mRNA molecule (**Figure 1**). However, in incomplete base-pair matching, mRNA translation will be suppressed (4, 5).

miRNAs can modulate 90% of protein-coding genes in several biological processes, such as proliferation, apoptosis, differentiation, immune cell lineage commitment, and maintenance of the immune system homeostasis (6). Some abnormalities in the immune system and the development of autoimmune diseases are highly related to the alteration of miRNAs’ transcription (7, 8). Studies indicated that these small molecules could be used as a biomarker to diagnose and monitor various autoimmune diseases. Moreover, targeting specific miRNAs could be another approach for autoimmunity treatment. However, it should be considered that each miRNA may have vast different mRNA targets. Therefore, the complicated interaction between specific miRNAs and the intact targeted genes has not been completely verified. Comprehensive genome studies indicated several single nucleotide polymorphisms (SNPs) in miRNAs and the expected miRNA target sites (9). In some cases, the alteration of miRNA function is induced by SNPs, possibly participating in disease development. miR-155 has been shown to target essential molecules involved in regulating the immune system. miR-155 is involved in different signaling pathways, including MAPK, insulin, Wnt, MAPK/Nuclear factor-κB (NFκβ), which highlights the importance of miR-155 in the different immune responses by targeting broad pathways (10). Several data have revealed that miR-155 may modulate immune cells, including dendritic cells (DCs), B cells, and T cells (11–13).

The precise discovery of immune regulation mechanisms by miRNAs has shed light on autoimmune diseases’ pathogenesis and helped us develop new therapeutic strategies against those diseases. This review article attempted to discuss the implications of miR-155 involvement in modulating the immune system and its contribution to autoimmune disease development by addressing both human and animal models.

## miR-155

### B Cells

miR-155 was first identified as a tissue-specific miRNA in an adult mouse (14, 15). one of the critical targets of miR-155 is the B cell integrating cluster (BIC), which is located on chromosome 21 (14, 16). Besides, mice with a mutation in miR-155 were diagnosed with B and T cell defects and antigen-presenting cell (APC) abnormal function. miR-155 deficient mice were identified with the reduced number of B cells’ germinal center. However, its overexpression results in an increased number of germinal center B cells (17, 18).

miR-155 may affect B cell maturation and isotype switching (19). The verexpression of miR-155 may cause pre-B cell lymphoma, and it seems that miR-155 may play an essential role in B cell function, and antagomiR-155 slowed pre-B cell tumors’ growth *in vivo* (20). It is important to mention that in miR-155 knockout animals, antibody production is reduced, and also, the number of germinal centers was decreased (17, 21). Additionally, the lack of miR-155 in B cells could reduce the secretion of IgG1 antibodies (Abs) and germinal center and extrafollicular response (19). By evaluating the gene expression profile of activated B cells, it has been observed that miR-155 controls the broad spectrum of genes with various functions, which are considered as the possible miR-155 target. For instance, the overexpression of the transcription factor Pu.1 in wild type B cells causes less IgG1 production, showing that Pu.1 is not regulated, which is the miR-155 deficient hallmark (19).

### T Cells

The process of T cell activation is controlled to provide a proper response toward infections and prevent autoimmune diseases. In miR-155 deficient mice, Th2 cells are the dominant phenotype, which causes c-Maf upregulation. C-Maf is a target for miR-155 and a potent transcription factor for IL-4 promoters. However, the Overexpression of this miRNA promotes the Th1 phenotype (17, 22). IFNγRα in CD4^+^ cells is a known target for miR-155 inhibit Th1 differentiation in miR155^-/-^ CD4^+^ cells. Besides, miR-155 deficient mice cannot polarize Th cells into Th17 follicular Th (FTH) cells (23–25).

Liu et al. reported that the miR-155-Peli1-c-Rel triad plays a significant role in TFH cells’ function and generation. Reduced proliferation of TFH cells, specifically at the late stages of differentiation and decreased expression of CD40 ligand (CD40L) on antigenic-specific CD4^+^ T cells, results from miR-155 deficiency (24). c-Rel is a protein regulated by miR-155 and Peli1 and essential in several B and T cell functions. c-Rel deficiency causes cellular proliferation defects in response to different stimuli (26). Plus, c-Rel deficient mice had several defects in germinal center response and antibody class switching. Upon T cell activation, this triad (miR-155-Peli1-c-Rel) is robustly induced by TCR and costimulatory molecules’ engagement. miR-155 modulates the expression level of Peli1 so that the Peli-1 allows the optimal level expression of c-Rel (26–28). The improper expression level of c-Rel has consequences. The lower level of c-Rel expression causes decreased germinal center response, diminished antibody production, and B and T lymphocyte activation defects. However, autoimmune diseases and lymphomas are the results of c-Rel Overexpression (26). Lack of miR-155 results in Othe overexpression of Peli1 in CD4+ T cells, which decreased the accumulation of c-Rel consecutively. This process finally led to the compromised expression of CD40L on CD4+ T cells and impaired proliferation of antigen-specific CD4+ T cells at the late TFH cell differentiation stage (24).

### Dendritic Cells (DC)

miR-155 expression is induced during immune cells’ activation, suggesting that this miRNA has a pivotal role in the immune system. miR-155^-/-^ results in immune-compromised mice. Upregulation of miR-155 is observed during DCs’ activation, and it has been reported that miR-155 is an essential factor for DC maturation. Mature DCs from miR-155 deficient mice exhibited functional and phenotypic defects. These defects in DCs are such as typical DCs morphology, decreased in the upregulation of costimulatory molecules, especially CD40 and CD86, robust decreased ability for antigen-specific CD4^+^ T cell activation and proliferation (17).

c-Fos is a transcription factor whose expression is negatively correlated with miR-155. During human Mo-DC and several subtypes of mice DC activation and maturation, the miR-155 expression level is increased; however, the c-Fos mRNA level is downregulated. Moreover, in miR-155 deficient mice, the c-Fos expression level is increased. c-Fos is a direct target for miR-155 since c-Fos mRNA has two binding sites in its 3’UTR. Therefore, it is concluded that during DC maturation, c-Fos expression is targeted and silenced by miR-155 mediated mechanism. However, it is not clear whether this mechanism is specific to DC (29–32).

### Macrophages

In response to tissue environment, different types of macrophages can be polarized. For instance, in bacterial infection, Th1, ILC1-derived IFNγ, and Toll-like receptor ligand (TLRL) induce proinflammatory macrophages (M1) that protect the immune system and can results in chronic inflammation. On the other hand, IL-10, transforming growth factor beta (TGF-β), glucocorticoids polarize the alternative macrophages (M2) which mediate the immune system resolution and restore the hemostasis. Different components, including miR-155 modulate macrophages polarization. Upon TLR/IFN-γ, miR-155 is induced in monocyte and macrophages. miR-155 defienct mice were reported witg decreased proinflammatory cytokines upon LPS stimulation. miR-155 blocks the polarization of M2 macrophages. By targeting different pathways, miR-155 inhibits STAT-6-driven antiinflammtory macrophage phenotype. Besides, miR-155 supresses TGF-β signaling pathway molecule Smad2 and prevents the development of repairment (33).

## miR-155 and Autoimmune Disease

In autoimmune diseases (AD), immunological tolerance is disrupted, and the immune system cannot distinguish self from non-self (34). Alteration of miR-155 was detected in human and animal models of various autoimmune diseases, including rheumatoid arthritis (RA), multiple sclerosis (MS), systemic sclerosis (SSc), systemic lupus erythematosus (SLE), and so on.

### Systemic Lupus Erythematosus

Systemic lupus erythematosus (SLE) is a multiorgan autoimmune disease that causes inflammation in the connective tissues (35). The worldwide incidence of SLE is higher in females and reproductive ages (36). In SLE, it has been shown that sex hormones, genetics, and environmental factors are involved in the dysregulation of the innate and adaptive immune system that can influence the disease onset (37).

**Figure 2:**
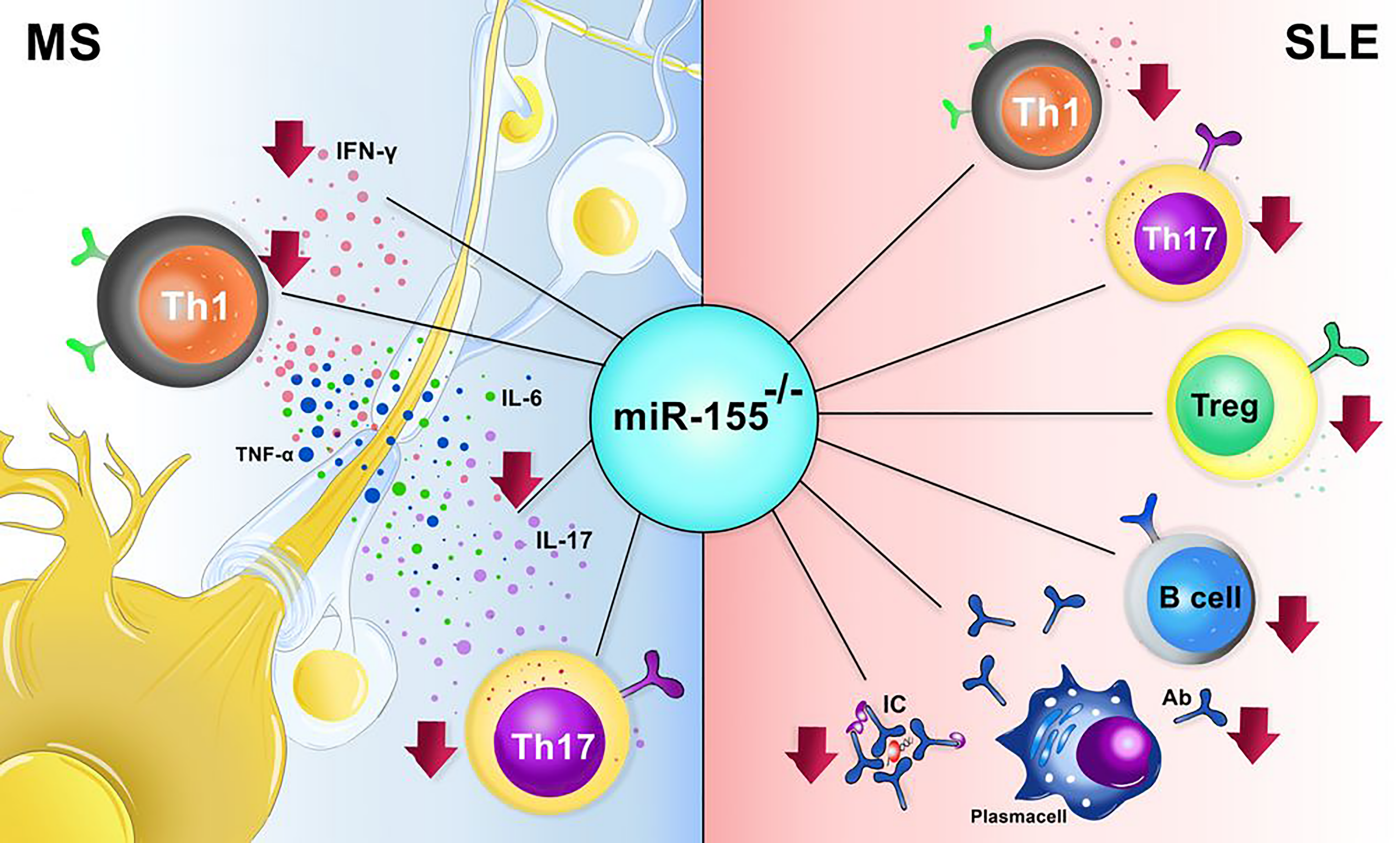
The effect miR-155^-/-^ in animal models of systemic lupus erythematosus (SLE) and multiple sclerosis (MS). In MS the absence of miR-155 results in decreased number of Th1, Th17 and also decrement procution of IL-17, IL-6, TNF-α, and IFN-γ. In SLE, lack of miR-155 causes a marked reduction in Th1, Th17, Treg, B cells, and plasma cells.

In the animal model of SLE (Pristane; PIL), deletion of miR-155 decreased the number of helper T (Th) 17 cells (38). Further studies indicated the reduction of IFN-γ-producing Th1 cells in miR-155 deficient pristane-induced lupus (PIL)^-/-^ mice compared to PIL^+/+^ mice (39). Surprisingly, in contrast to other studies that detected a moderate amount of IL-4 producing cells in miR-155^-/-^ mice, Leiss et al. discovered less IL-4 producing lymphocytes in miR-155^-/-^ PIL animals than in wild type PIL mice (39). Thymic development is interrupted in the PIL mice, and the Treg cells are arrested and however, in miR-155^-/-^ mice, the number of CD4^+^FOXP3^+^ Treg cells is reduced, but the tolerance is maintained due to the presence of peripheral tolerance (**Figure 2**) (40–42).

As mentioned already, miR-155 can regulate B cell function and, therefore, miR-155 can participate in autoantibody production in SLE. Several studies have indicated reducing autoantibody production in miR-155 knocked out MRL-lpr lupus-prone mice with amelioration in the kidney inflammation (43). Moreover, studies revealed protection against pulmonary hemorrhage in miR-155 deficient and PIL mice. Besides, administration of miR-155 antagomir alleviates pulmonary hemorrhage induced by pristane (44). Lashine et al. pointed out that overexpression of miR-155 led to higher expression of interleukin (IL)-2 in peripheral blood mononuclear cells (PBMCs) based on suppression of negative regulator of this cytokine, namely protein phosphatase two catalytic subunit alpha (PP2Ac). Overexpression of miR-155 could be a possible explanation for increased expression of IL-2 by less expression of its regulator (PP2Ac) in juvenile SLE disease (45).

Sphingosine-1-phosphate receptor 1 (S1PR1) is another target of miR-155, and it was reported to be decreased at transcriptional and translational levels in SLE patients. S1PR1 plays a role in the pathogenesis of SLE. Knocking out the miR-155 in Fas^lpr/lpr^ mice ameliorated the disease symptoms, and reduced the serum level of immunoglobulin (Ig) G, IgM, and diminish the immune complex deposition in the kidneys of treated mice (**Figure 2**) (46). However, a controversial result was obtained from different studies based on different targets of miR-155 in SLE; Overexpression of S1PR1 was detected in the miR-155^-/-^ Fas^lpr/lpr^ mice, whereas microarray analysis indicated the reduction of S1PR1 in SLE patients (46, 47). S1PR1 gene expression might be associated with SLE pathogenesis and considered as a therapeutic option in SLE treatment. Since the role of S1PR1 in disease-related mechanisms has not been fully understood, more investigation concerning the role of this gene in the pathogenesis of SLE is required.

### Rheumatoid Arthritis

Rheumatoid arthritis (RA) is a systemic inflammatory autoimmune disease that affects more than 1% of the world population and causes severe disability in patients (21, 48). This disease’s clinical manifestations include synovial inflammation and hyperplasia, autoantibody production, cartilage, and bone destruction, including skeletal, cardiovascular, pulmonary disorders. Genetic, epigenetic, and environmental factors together play a role in the induction of disease. However, RA pathogenesis’s exact mechanism has not yet been fully understood (21, 49).

Several studies have evaluated miRNAs in RA to find a new biomarker for RA or establish a new therapeutic strategy. Stanczyk et al. indicated the progressive effect of miR-155 in RA development (50, 51). The increment of miR-155 has been reported in different cell types or tissues of RA, such as synovial tissue (51–53), CD68^+^ synovial macrophages (50, 52–54), RA synovial fluids (RASFs) (51, 55), synovial fluid CD14^+^ cells (51, 54), PBMCs (56–58), and whole blood of RA patients (58). However, the level of miR-155 was reduced in the sera of RA patients (52, 59, 60).

**Figure 3:**
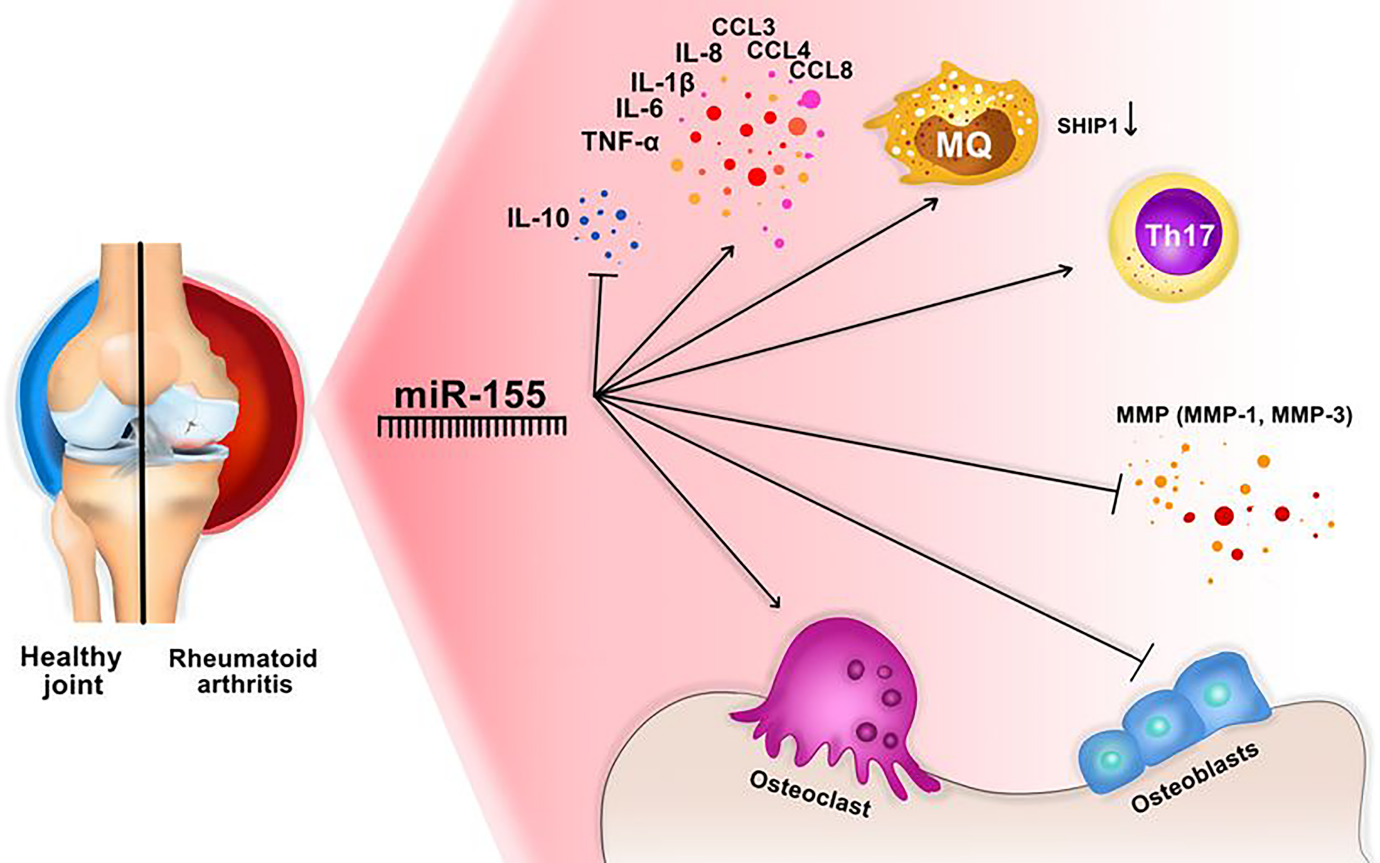
miR-155 might have an important role in RA development. This miRNA activates Osteoclasts activation, Th17 cells, macrophages, and inflammatory cytokines, and blocks osteoblast production, and inhibitory cytokines, including IL-10. In addition, MMP production (especially MMP-1 and MMP-3) are decreaced through downregulation of IKBKE.

It is shown that miR-155 can affect the different types of cytokines in RA. For instance, the pleiotropic cytokine, namely the tumor necrosis factor α (TNF-α), is produced by various cells, such as monocytes, macrophages, B cells, T cells, and fibroblasts. TNF-α is highly increased in RA that causes bone distraction, pain, and inflammation (50, 61). It was shown that an elevated level of miR-155 correlates with the upregulation of TNF-α and IL-1β and downregulation of SOCS in RA (50, 62). Spoerl et al. discovered that inhibition of miR-155 was associated with suppression of osteoclasts and increased numbers of osteoblasts (**Figure 3**) (14, 63). Further, Wu et al. reported that TNF-α could cause the Overexpression of miR-155, and knocking down the miR-155 could reduce the TNFα- mediated inhibition of bone morphogenic protein 2 (BMP-2). Furthermore, miR-155 can modulate TNF-α regulated osteogenic differentiation by targeting SOCS1 (14, 64).

Overexpression of miR-155a could also change the expression level of other cytokines and chemokines such as IL-1β, IL-6, IL-8, TNF-α, chemokine ligand (CCL) 3, CCL4, CCL5, CCL8 and downregulate the synthesis of IL-10 and C-C chemokine receptor type 2 (CCR2) in synovial fluid of RA patients (21, 54). In addition, miR-155 is required for the homeostasis of IL-17 producing cells (**Figure 2**) (50, 52, 54, 56).

It is also proposed that miR-155 is required for homeostasis and function of Treg cells and IL-17 producing cells (50, 52, 54, 56). Another study evaluated the role of miR-155 in the collagen-induced arthritis (CIA) mouse model, which showed that the CIA could not develop arthritis in miR-155^-/-^ mice (50, 65). Autoreactive B and T cells play a crucial role in CIA, and the absence of miR-155 prevented the generation of pathogenic autoreactive B and T cells in CIA, which was associated with a significant reduction of local bone destruction and antigenic-specific Th17 cells suppression (65, 66). Therefore, based on the inhibition of autoreactive B and T cells and less bone destruction in miR-155 deficient mice, miR-155 might be considered a potential target for RA treatment.

Already it was mentioned that miR-155 is detected in synovial fluid of RA patients, and its Overexpression was detected in synovial membrane and macrophages of synovial fluid in RA patients. Simultaneously the elevation of miR-155 was associated with the reduction of the Src homology 2‐containing inositol phosphatase‐1 (SHIP‐1) expression, which is an inhibitor of inflammation (**Figure 3**).

One of the important targets of miR-155 is the inhibitor of nuclear factor kinase subunit epsilon (IKBKE) (21, 67). IKBKE can induce matrix metalloproteinase (MMP) expression and causes joint damage in arthritis (21, 68). MMPs, especially MMP3, have been shown to be involved in the proliferation and invasion of RA-FLS (Fibroblast like synovium) (21, 69). Therefore, miR-155 may limit the production of MMPs through the downregulation of IKBKE and reduced the invasion of FLS through MMPs (**Figure 3**) (21). To sum up, miR-155a plays a different role in RA progression, which could be used as a target in RA therapy.

This result might indicate the role of M1 macrophages in the process of RA pathogenesis (51, 70). Besides, Kurowska Stolarska et al. revealed that miR-155 expression level is upregulated in synovial macrophages and monpcytes of patients diagnosed with RA. This Overexpression promotes proinflammatory cytokiens production (54).

### Multiple Sclerosis

Multiple sclerosis (MS) is an autoimmune disease of the central nervous system (CNS), and its prevalence in young women is higher than in men (71, 72). Scientists detected the upregulation of mir155 n paraffin and frozen sections of white matter lesions from MS patients (73). Moreover, Noorbakhsh et al. discovered the elevation of miR-155 in the cerebral white matter of relapsing-remitting, primary progressive, and secondary progressive MS patients (74). In addition, the Overexpression of miR-155 was found in various cells of the immune and nervous system, including myeloid-derived macrophages, microglia, T and B cells, and astrocytes, besides resident brain cells (73, 75). Furthermore, Overexpression of miR-155 was discovered in a neurovascular unit of active lesions from MS brain samples (76–78). Interestingly, the combination of miR-155, miR-146a, and miR-142-3p increment had a predictive value for diagnosing 88% of MS cases (78). The Overexpression of miR-155 was associated with the overproduction of IL-17, IFN-γ, TNF, and IL-6 in sera of patients diagnosed with MS, suggesting that the elevation of miR-155 may occur only during inflammation (**Figure 2**) (75, 79).

A cohort study in the Egyptian population found that the TT genotype and the T allele in miR-155 polymorphism (rs767649 A> T) were related to the higher prevalence of MS in women, but not in men. The genotype of miR-155 rs767649 AT/TT was associated with secondary progressive MS (80).

#### Experimental Autoimmune Encephalomyelitis (EAE)

Experimental autoimmune encephalomyelitis (EAE) is an animal model of MS. An emulsified myelin basic protein (MBP) or myelin oligodendrocyte glycoprotein (MOG) with Freund’s adjuvant and pertussis toxin were used to develop this animal model of the disease. DCs actively present MBP or MOG to CD4^+^ T cells in lymph nodes that causes infiltration of CD4^+^ Th1 and Th17 cells, B cells, CD8^+^ T cells, and innate immune cells to the CNS and leading to tissue damage (81). The function of miR-155 was identified in miR-155 knockout mice, which were shown to be tremendously resistant to develop MOG35-55 peptide-induced EAE. The onset of disease in miR-155^−/−^ mice was delayed, and the disease severity and paralysis were reduced in comparison to wild-type mice were observed (12, 82). The analysis of brain histology from miR-155^-/-^ EAE mice revealed less inflammation and less demyelination (12, 82). The absence of miR-155 in the EAE model caused a reduction of Th1 and Th17 in the spleen, lymph nodes, and the CNS (12, 83). In an *ex vivo* study, the miR-155 knocked out mice showed less production of IFN-γ and IL-17 upon stimulation with antigen, demonstrating the functional defect in Th1 and Th17 cells. In another adoptive study, transfer of miR-155^+/+^ CD4^+^ T cells into Recombination activating gene 1 (RAG1)^−/−^ mice led to EAE progression and increased disease severity in comparison to mice receiving miR-155^−/−^ CD4^+^ T cells (12). Moreover, Jiang et al. suggested that antigen-specific CD4^+^ T cells in EAE could increase miR-155 production upon contact with MBP (84). Additionally, the role of miR-155 in driving Th1 and Th17 responses were revealed when a locked nucleic acid (LNA)-miR-155 oligonucleotide (herein called “antagomir”) was delivered before or during EAE induction in mice (79, 82). The administration of miR-155 antagomir reduced the IFN-γ and IL-17 production in antigen-specific CD4^+^ T cells in mice CNS (82). Administration of miR-155 in EAE mice enormously increased the severity of inflammation, demyelination in the spinal cord, number of Th1 and Th17 cells. Furthermore, increased IL-17 and IFN-γ production was observed in the spleen, lymph nodes, and CNS of mice (79). Taken together, all the above studies suggest that the Overexpression of miR-155 in EAE increases the functionality of antigen-specific Th1/Th17 cells.

O’Connell and colleagues showed that miR-155 targets the negative regulator of Th17 differentiation, namely transcription factor Ets1 (85). Moreover, they indicated the elevation of Ets1 and lack of Th17-related cytokines in miR-155^−/−^ mice. In other words, the axis of Ets1/miR-155 is required for normal Th17 expansion and cytokine production in EAE (85). Furthermore, Escobar et al. performed transcriptome analysis and indicated that miR-155 could regulate the chromatin structure and epigenetic changes in Th17 cells (23). Besides, they identified that RNA binding protein Jarid2 was increased in miR-155^-/-^ mice, which can reprogram the epigenome of Th17 cells *via* H3K27 methylation that causes *IL-22* gene silencing. This cytokine is necessary for Th17 differentiation (23). In the absence of miR-155, Th17 differentiation and cytokine expression were interrupted, but it could be resorted by deletion of Jarid2 (23). Another study demonstrated the role of miR-155 in cell migration through heme oxygenase-1 (HO-1) repression. EAE was restored in miR-155^-/-^ mice by HO-1 inhibitor ZnPP injection (86). Mycko et al. discovered the increased expression of miR-155-3p in CD4^+^ T cells isolated from CNS at the peak of EAE, whereas miR-155-5p is not highly expressed at this time point (87). In fact, they showed that miR-155-3p could drive the Overexpression of RORα and IL-17 in comparison to miR-155-5p, which could particularly upregulate the Th17 differentiation (87). Taken together, miR-155 might be responsible for cytokine expression in Th1 and Th17 and also cell migration in animal models of EAE.

In order to reduce the symptoms of MS disease, immunomodulatory drugs that suppress the recruitment of immune cells to the CNS are widely in use. Medications mainly act on the immune system as immunosuppressant drugs (natalizumab, fingolimod, mitoxantrone), which are able to lessen the activity of the immune cells [like IFN-β, glatiramer acetate, dimethyl fumarate (DMF)] or suppress cell proliferation (like teriflunomide, alemtuzumab, ocrelizumab) (88). Whereas miR-155 is elevated in MS patients and in EAE, it is noteworthy to study the role of the aforementioned drugs on miR-155 expression. IFN-β and glatiramer acetate did not affect the expression of miR-155, but they declined other miRNAs expression (78, 89). Nevertheless, glatiramer acetate decreased miR-155 expression in urine-isolated exosomes in EAE (90). DMF showed a reduction of Th1/Th17 subsets, an elevation of Th2 subsets, and a shift from M1 toward M2 macrophages (91, 92). DMF can prevent microglia and astrocyte inflammation *in vitro* and EAE animal models (93, 94). The patients who received DMF showed a reduction of miR-155 expression in monocytes (95). Natalizumab could decrease the expression of miR-155 in PBMCs and monocytes of MS patients, which may lead to downregulation of IL-17, IFN-γ, and TNF (76, 95). Fingolimod can remarkably reduce miR-155 expression in human monocytes (76). Overall, we suggest that treatment with several popular drugs would decrease the expression of miR-155, which could be considered as a target for MS therapy.

### Systemic Sclerosis

Systemic sclerosis (SSc) is an autoimmune disease characterized by excessive deposition of the extracellular matrix, vasculopathy of small vessels, and autoantibodies production (96). This disease’s manifestations vary in different patients; however, skin thickening and different internal organs’ involvement are the main manifestations (97). Interstitial lung disease (ILD) is known to be the leading cause of mortality in SSc patients (98–100). The prevalence of ILD is high in SSc patients, and approximately 15–30% of patients will develop severe lung fibrosis (101, 102).

Elton et al. showed that dysregulated miR-155 in ILD was strongly associated with progressive lung disease. In addition, this specific miRNA is highly expressed in activated monocytes/macrophages from SSc patients (103). Moreover, the expression of miR-155 is highly correlated with the expression of profibrotic genes, such as secreted phosphoprotein 1/osteopontin (SPP1) and periostin (POSTN). Furthermore, the expression of miR-155a is increased in PBMC of SSc patients with ILD (104). Interestingly, lung fibrosis was observed to be less developed in miR-155^-/-^ mice and the survival of mice was higher than miR-155^+/+^ mice (104).

Yan et al. indicated the inhibition of alternatively activated macrophages (M2) in the lungs of miR-155^-/-^ mice (105). The inhibition of miR-155 through the antagomiR-155 resulted in less skin thickness in the bleomycin-induced SSc animal model (105). Moreover, intratracheal administration of bleomycin increased the expression of miR-155 in the lungs of mice, which directly correlated with lung fibrosis (106). Taken together, the increment of miR-155 is involved in lung fibrosis in SSc patients.

### Behçet’s Disease

Behçet’s disease (BD) is an autoinflammatory disease that affects several organs and causes uveitis, oral aphthae, skin lesions, and genital ulcers (107, 108). In a study done by Zhou et al., the decreased expression of miR-155 was found in PBMC, DCs, and CD4^+^ T cells of BD patients. Also, induced Overexpression of miR-155 in DCs inhibited the expression of IL-1β, IL-6 and promoted the IL-10 expression, and Overexpression of miR-155 in CD4^+^ T cells inhibited the expression of IL-17, which would suggest that miR-155 is a negative regulator of inflammatory cytokines in BD (109).

### Type 1 Diabetes

Type 1 diabetes (T1D) is an autoimmune disease mediated by activation of T cells and macrophages and the production of inflammatory cytokines that further activate the immune system and cause insulitis, and β cell damage results in beta-cell damage and reduction of insulin production (110, 111). Garcia-Diaz et al. reported the elevation of miR-155 and reduction of miR-146a in PBMCs of T1D patients (112). However, controversial data were achieved from the investigation of Assmann et al., which indicated the protective role of miR-155 and miR-146a in TID Based on linkage analysis of miR-155 rs767649 and miR-146a rs2910164 polymorphisms. They concluded that these polymorphisms could reduce miRNA expressions, which led to the activation of nuclear factor (NF)-κB and higher production of inflammatory cytokines in the pancreas (113).

### Primary Immune Thrombocytopenia

Primary immune thrombocytopenia (ITP) is an autoimmune disease mediated by autoantibodies against platelets’ surface antigen (gpIIb-IIIa) which activates opsonization and phagocytosis. The disease is manifested by bleeding and reduction of platelet count (114). Studies indicated the higher expression of miR-155 in PBMCs of ITP patients positively correlated with the reduction of platelet count. Also, decreased SOCS1, IL-4, IL-10, and TGF-β in mRNA levels were detected. Furthermore, the increment of IL-17A in plasma of ITP patients correlated with miR-155 Overexpression. Therefore, these data suggest that miR-155 might be involved in the pathogenesis of ITP by modulating cytokines and targeting SOCS1 (115).

In contrast, different experiments revealed that the number of Treg lymphocytes number in the peripheral blood of patients diagnosed with ITP is significantly lower compared to healthy individuals, and the inhibitory function of these cells is significantly attenuated. It was reported that several miRNAs, including miR-155–5p, might play a major role in regulating the growth and function of Treg cells (116). several results indicated that miR-155-deficient mice have fewer Treg cells in the thymus and periphery due to growth defects. However, an elevated level of miR-155 could play a role in increasing the inhibitory function of Treg cells (40). These findings suggest that miR-155 is involved in the development and function of Treg cells. Further, miR-155 has been significantly reduced in Treg cells, which may cause Treg dysfunction and the pathogenesis of ITP (116).

### Inflammatory Bowel Disease

Inflammatory bowel disease (IBD) is a chronic inflammatory disease of the intestinal tract with unknown etiology. However, genetic and environmental factors play a role in the pathogenesis of the disease (117–119). The infiltration of various cells, including Th cells, macrophages, and neutrophils to the mucosal part and release of inflammatory mediators cause inflammation and changes the intestine’s architectural structure in IBD. In the experimental model of IBD, it was observed that CD4 cells, especially Th1 cells, have a high capacity in the disease induction and damaging the intestine (120–122). In addition, in patients and animal models of colitis, the systemic level of TNF-α, IFN-γ, IL-6, and IL-12 is significantly increased, which might be responsible for inflammatory status in IBD (123–126). Singh et al. discovered fewer symptoms (minor change in body weight and no diarrhea or blood in feces), decreased numbers of Th1/17 cells, macrophages, and DCs as well as the reduced amount of inflammatory cytokines such as TNF-α, IFN-γ, IL-6, and IL-12 in miR-155 deficient animal model of IBD. Moreover, miR-155 might have a crucial role in the devolvement of dextran sulfate sodium (DSS)-induced colitis in mice (127).

### Sjogren’s Syndrome

Sjogren’s syndrome (SS) is an autoimmune disease characterized by dysregulation of salivary, lachrymal of eyes, and mouth glands, which results in dryness of eyes (keratoconjunctivitis) and mouth (xerostomia) (128–136). The exact disease pathogenesis is unknown; however, the presence of type I IFN, B cell-activating factor (BAFF), and IL-12 indicate an interaction between innate and adaptive immune responses in SS pathogenesis. Moreover, the involvement of the activated Th1, Th17, and Natural killer (NK) cells was discovered in this disease (131, 134). Downregulation of miR-155 was detected in PBMCs from patients with SS that correlated with visual analog scale (VAS) score for dry eyes, indicating the involvement of miR-155 in the pathogenesis of SS (137). Interestingly, Le Dantec et al. discovered the Overexpression of miR-155 in Foxp3 positive infiltrating cells in these patients’ salivary gland and epithelial cells an ameliorating role of miR-155 in SS (138).

### Guillain–Barré Syndrome

Guillian–Barré syndrome (GBS) is a rare and acute autoimmune disease caused in response to primarily viral and bacterial infection. The infiltration of inflammatory cells and their responses attack peripheral nerves and results in demyelination of neurons, and provokes polyneuropathy (139). The disease is characterized by extremities’ weakness, paralysis of eye muscles, and the absence of tendon reflexes (140). Wang et al. detected the downregulation of miR-155 in PBMCs of GBS patients. Moreover, by silencing miR-155, they detected the elevation of Th1 cytokines *in vitro.* Therefore, they proposed a protective role of miR-155 in GBS (141).

### Ankylosing Spondylitis

Ankylosing spondylitis (AS) is a chronic autoimmune disease that mainly affects the spine and sacroiliac joints (142). The disease is known by immoderate bone formation, with syndesmophytes as the typical lesion (143, 144). Qian et al. observed the elevation of miR-155 in the serum of AS patients. Moreover, they suggest that this upregulation can be used as a biomarker for disease prognosis (145).

### Vitiligo

Vitiligo is an acquired autoimmune disease with an unknown etiology. Vitiligo is characterized by the demolishment of melanocytes (146). It has been proposed that genetic and environmental factors can play a role in the disease etiopathogenesis. The physical symptoms are usually rare, except for depigmented macules on the skin (147, 148). The dysfunctionality of melanocyte, keratinocyte, and alteration of keratinocytes in depigmented skin can play a major role in the disease manifestations (149). Šahmatov et al. observed the elevated level of miR-155 in stratum basale (where melanocytes and proliferating keratinocytes are located) as well as in stratum spinosum of the epidermis of patients with vitiligo. Moreover, the Overexpression of miR-155 was associated with Overexpression of inflammatory cytokines, such as TNF-α, IFN-α, IFN-γ, and IL-1β in melanocytes and keratinocytes. Overexpression of miR-155 resulted in inhibition of the melanocyte differentiation and melanogenesis genes, such as tyrosinase-related protein 1 (TYRP1), tyrosine 3-monooxygenase/tryptophan 5-monooxygenase activation protein epsilon (YWHAE), syndecan binding protein (SDCBP), and sex-determining region Y-Box 10 (SOX10) in melanocytes, and YWHAE in keratinocytes. Furthermore, Overexpression of miR-155 could alter the expression of SOCS1, interferon regulatory factor 1 (IRF1), and interferon-induced transmembrane protein 1 (IFITM1) in melanocytes and keratinocytes (150). Hence, it was suggested that miR-155 played a major role in the pathogenesis of Vitiligo.

### Grave’s Disease

Grave’s disease (GD) is an organ-specific autoimmune disease represented by diffuse goiter and hyperthyroidism. GD is diagnosed by high levels of free triiodothyronine (FT3), free thyroxine (FT4), and the presence of thyroid-specific autoantibodies like anti-thyroglobulin antibody (TGAb), anti-thyroperoxidase antibody (TPOAb), and anti-thyrotropin receptor antibody (TRAb). Treg cells play a pivotal role in the development of the disease (151, 152). Some studies indicated that GD patients had a lower expression level of Foxp3 and decreased CD4^+^CD25^+^ Treg cells (153–156). Still, the exact mechanism of Treg cell dysfunctions is obscure (157). Zheng et al. claimed that miR-155 had a vital role in GD development by modulating Treg cells. Moreover, the level of miR-155 in sera of GD patients was lower than in healthy controls. The miR-155 serum level reduction was more notable in women (157). Since Foxp3 is a target of miR-155 (40, 158), decrement of miR-155 could impair Treg cell development in GD patients (157).

### Graves’ Ophthalmopathy

Graves’ ophthalmopathy, also known as thyroid eye disease (TED), dysthyroid/thyroid-associated orbitopathy (TAO), Graves’ orbitopathy (GO), is an autoimmune disease of the orbit and periorbital tissues. Graves’ ophthalmopathy is caused by an inflammation in the orbital connective tissue due to increment of inflammatory cytokines, especially IL-6, elevated orbital volume due to overproduction of glycosaminoglycan, and enhanced adipogenesis (159, 160). However, the exact mechanism underlying the pathogenesis of the disease is still obscure (161). Various studies have implied the CD4 cells as major culprits in disease pathogenesis. Also, current studies focused on the role of miRNAs in disease pathogenesis. Histochemical examination of the patient’s orbit revealed the infiltration of lymphocytes. Furthermore, fibroblasts play an important role in Graves’ ophthalmopathy occurrence and development (162, 163). Li et al. reported increased expression of miR-155 in CD4^+^ cells and fibroblasts from TED patients. Overexpression of miR-155 caused ocular inflammation in target cells (CD4^+^ cells & ocular fibroblasts) (164).

### Psoriasis

Psoriasis is an autoimmune disease mediated by T cells and causes skin inflammation. The disease is characterized by scaly debris and invasive erythema, along with different degrees of itching (165). Xu et al. detected significant upregulation of miR-155 in tissues of psoriasis patients. They proposed that verexpression of miR-155 promoted T cell proliferation and eliminated the apoptosis through phosphatase and tension homolog (PTEN) signaling pathway (166).

Alatas et al. discovered that expressions of miR-155-5p along with other miRNAs significantly increased in patients diagnosed with psoriasis compared with the control group. However, disease severity was not correlated with miRNAs (167).

Stimulation of TNF-α enhances miR-155 expression independently of dosing, and miR-155 suppressor significantly reverses TNF-α-induced inhibition at the GATA3 protein level and enhances the production of CXCL8 and IL-6. miR-155 can inhibit the GATA3 expression by targeting 3’UTR, while GATA3 can induce IL-37 transcription by targeting its promoter region. Overexpression of miR-155 decreases IL-37 protein and enhances the production of CXCL8. GATA3 Overexpression may significantly reduce the miR-155 overexpression effects. Unlike GATA3, the expression of miR-155 significantly increased in the tissues of psoriasis lesions and is negatively associated with IL-37 and GATA3. Briefly, the miR-155/GATA3/IL-37 axis regulates the production of CXCL8 and IL-6 by stimulating TNF-α to affect the progression of psoriasis. However, miR-155/GATA3/IL-37 may be an available option for psoriasis treatment, which requires additional clinical studies (168).

### Myasthenia Gravis

Myasthenia Gravis (MG) is an antibody-mediated autoimmune disease. These antibodies are against neuromuscular junction (NMJ) proteins and cause neuromuscular transmission impairment. Clinical manifestations range from extensive weakness and ocular symptoms to failure in the respiratory system (169). Overexpression of miR-155 was shown in PBMCs from MG patients. Knocking out the miR-155 in the experimental myasthenia gravis (EAMG) model resulted in decreased autoantibodies against acetylcholine receptors as well as the disease severity. Therefore, it was suggested that miR-155 might primarily affect the B cells in MG patients (170, 171).

### Idiopathic Inflammatory Myopathies

The idiopathic inflammatory myopathies (IIMs) are rare autoimmune disorders characterized by skeletal muscle inflammation. Patients are often become weak and are disabled with poor quality of life (170). Five miRNAs, including miR-155, have been found to be upregulated across ten primary muscle disorders, including IIMs (172).

## Concluding Remarks

The present review was conducted to discuss the available data associated with miR-155 expression and function alterations in the immune system from human autoimmune disease as well as related animal models. miR-155 exerts a significant impact on the homeostasis and development of the immune system. The aberrant function and expression of miR-155 have been related to several human and animal models of autoimmune disease, suggesting that the appropriate regulation of miR-155 may confer a key approach in preventing autoimmune diseases. Nonetheless, experimental and functional research is required to verify and establish the causal relationship between the development of autoimmune diseases and aberrantly-expressed miR-155. In addition, mechanisms contributing to the aberrant expression of miR-155 and the effect of other regulating factors of miR-155 need to be clarified. Given the contribution of miR-155 to autoimmune diseases, diagnostic and therapeutic strategies are recommended to be taken using its great potential for developing autoimmune diseases. Ultimately, much attention is needed to be paid to improving technologies required for the *in vivo* delivery of miRNA mimics or inhibitors targeting miR-155 as a therapeutic purpose.

## Author Contributions

SP, MM, FV, and AK were contributed in drafting the manuscript. NM supervised and revised the final version of the manuscript. All authors contributed to the article and approved the submitted version.

## Conflict of Interest

The authors declare that the research was conducted in the absence of any commercial or financial relationships that could be construed as a potential conflict of interest.
